# Mitigation of disease- and treatment-related risks in patients with psoriatic arthritis

**DOI:** 10.1186/s13075-017-1265-5

**Published:** 2017-03-20

**Authors:** Martin Bergman, Amy Lundholm

**Affiliations:** 1 Taylor Hospital, 8 Morton Avenue, Suite 304, Ridley Park, PA 19078 USA; 2 Lankenau Medical Center, Wynnewood, PA USA

**Keywords:** Psoriatic arthritis, Disease-specific risks, Treatment-related risks, Psychological risks, Comorbidities, Quality of life, Risk, Review

## Abstract

Psoriatic arthritis is a part of the family of diseases referred to as spondyloarthropathies, a diverse group of chronic inflammatory disorders with common clinical, radiographic, and genetic features. Peripheral arthritis is the most common symptom of psoriatic arthritis and patients also frequently experience involvement of the entheses, spine, skin, and nails. Due to the diverse clinical spectrum of disease severity, tissues affected, and associated comorbidities, the treatment of psoriatic arthritis can be challenging and it is necessary to mitigate risks associated with both the disease and its treatment. These risks include disease-specific, treatment-related, and psychological risks. Disease-specific risks include those associated with disease progression that can limit functional status and be mitigated through early diagnosis and initiation of treatment. Risks also arise from comorbidities that are associated with psoriatic arthritis such as cardiovascular disease, obesity, diabetes mellitus, and gastrointestinal inflammation. Patient outcomes can be affected by the treatment strategy employed and the pharmacologic agents administered. Additionally, it is important for physicians to be aware of risks specific to each therapeutic option. The impact of psoriatic arthritis is not limited to the skin and joints and it is common for patients to experience quality-of-life impairment. Patients are also more likely to have depression, anxiety, and alcoholism. This article reviews the many risks associated with psoriatic arthritis and provides guidance on mitigating these risks.

## Background

Psoriatic arthritis (PsA) is a member of the spondyloarthropathies (SpA) family of diseases, a diverse group of chronic inflammatory rheumatic disorders with common clinical, radiographic, and genetic features [1, 2]. There are a wide variety of clinical and anatomic characteristics that distinguish PsA from other chronic rheumatic diseases such as rheumatoid arthritis (RA) and other forms of SpA [3, 4]. The primary regions affected in patients with PsA are the peripheral joints, entheses (connective tissue between tendon or ligament and bone), and axial sites, often in an asymmetrical pattern, in addition to psoriasis of the skin and nails [4, 5]. In addition to the larger joints, the small joints of the fingers, including the distal interphalangeal (DIP) joints—which are generally spared by RA—and toes are also typically involved in PsA [5]. Further, up to 43% of patients with PsA experience sacroiliitis (inflammation of the sacroiliac [SI] joint) in the spinal column [6]. Dactylitis (swelling of an entire digit) is also common in patients with PsA and is a marker of disease progression [7]. Although PsA is commonly associated with the presence of psoriasis, skin disease and joint inflammation do not always present concurrently [2].

The prevalence of PsA in the United States is 0.25% [8]. However, the prevalence of PsA in patients with psoriasis is substantially higher, with approximately 30% of individuals reported to have both conditions [9]. PsA is reportedly more common in women than in men compared with ankylosing spondylitis [5, 10]. Although it is important to recognize that there may be some degree of selection bias in this regard (due to conventional wisdom and teaching that women are unlikely to develop ankylosing spondylitis).

The differentiation of PsA from RA can be aided by certain clinical characteristics. Unlike RA, PsA is not associated with circulating autoantibodies [3, 11]. Patients with PsA are usually seronegative (absence of circulating rheumatoid factor) and a negative test for rheumatoid factor is one component of the ClASsification criteria for Psoriatic Arthritis (CASPAR) [2, 12]. Additionally, PsA and RA typically have distinct patterns of inflammatory joint damage, which can be distinguished through specific clinical and radiological changes. The clinical pathology of PsA typically presents in the distal interphalangeal and axial joints, with an asymmetrical distribution, whereas RA is primarily symmetrically distributed in the metacarpophalangeal and wrist joints [3]. However, PsA may also present as a symmetrical arthropathy or as an oligoarthropathy. The vascular pathology of PsA is distinct from RA and is characterized by a hypervascularized network of elongated, tortuous vessels [3]. Whereas RA primarily results in bone and cartilage resorption, PsA has both bone destruction and formation traits [13]. Both patients with RA and PsA experience erosion that leads to resorption of cortical bone, but additional formation of bony spurs is observed along insertion sites of entheses only in patients with PsA [13].

Like other forms of SpA, susceptibility to PsA is associated with the human leukocyte antigen B27 (HLA-B27) gene [2]. The strength of the association between HLA-B27 and disease susceptibility varies among different SpA subtypes as well as between ethnic groups and the presence of HLA-B27 in combination with other major histocompatibility complex class alleles may influence the pattern of axial or peripheral disease presentation [14].

The clinical spectrum of PsA is extremely diverse in both disease severity and tissues affected, and this disorder often occurs in conjunction with several associated comorbidities [4, 15]. The array of symptoms, coupled with the wide range in severity and disease course, presents difficult challenges for treatment [16]. Mitigation of risks associated with the disease itself, its treatment, and associated comorbidities are all important considerations when managing patients with PsA (Table 1). This review will discuss and highlight the many risks associated with PsA, with the aim to help improve awareness of risks among physicians and to provide guidance in mitigating these risks.

**Table 1:** PsA risk framework

Disease-related risks (including functional concerns)
• Assessment of symptoms (pain, stiffness, swelling, rash) ○ Physical examination ○ Joint examination ○ PASI • Functional assessment ○ Imaging (X-rays, MRI) • Quality of life (social interaction, sexual health, body image) ○ SF-36 subscales ○ EuroQoL-5 dimension ○ PsAQoL ○ Pain Disability Index ○ PsAID ○ Work ability • Documentation of extra-articular manifestations and/or comorbidities • Poor balance/risk of falls ○ Fractures
Treatment-related risks
• Contraindications ○ NSAIDs in patients with IBD, CV disease • Adverse events ○ Liver damage with methotrexate (obese patients particularly at risk) • Poor compliance/persistence ○ Reduced efficacy of biologics • Routine laboratory monitoring with biologics • Immunogenicity with biologics
Psychosocial risks
• Mental health (particularly depression, but also anxiety) ○ SF-36 subscales ○ PsAID ○ DASS-21 • Alcohol abuse • Self-esteem issues (especially in younger patients) • Social participation ○ PsAID

*CV* cardiovascular, *DASS-21* Depression and Anxiety Stress Scale, *IBD* inflammatory bowel disease, *MRI* magnetic resonance imaging, *NSAIDs* nonsteroidal anti-inflammatory drugs, *PASI* Psoriasis Area and Severity Index, *PsAID* Psoriatic Arthritis Impact of Disease, *PsAQoL* Psoriatic Arthritis Quality of Life Questionnaire, *SF-36* Short-form 36

## Disease-specific risks

### Risks associated with disease progression

Early diagnosis and treatment of PsA is important because the disease not only causes functional impairment over time, but also may increase the risk of mortality in affected patients [15]. Unfortunately, early detection methods for PsA remain limited and the disease is often underdiagnosed due to symptoms going unrecognized [15]. Common initial symptoms include arthritis in the upper and lower limbs (Table 2).

**Table 2:** First signs and symptoms attributable to psoriatic spondyloarthritis [74]

First signs and symptoms, n (%)	Psoriatic spondyloarthritis		
	≤2 years (*n* = 51)	>10 years (*n* = 187)	*P* value
Low back pain	13 (26)	31 (17)	0.15
Sacroiliac syndrome	6 (12)	17 (9)	0.59
Neck pain	1 (2)	14 (7)	0.20
Dactylitis	5 (10)	17 (9)	0.79
Arthritis, lower limbs	29 (57)	131 (70)	0.08
Arthritis, upper limbs	27 (53)	106 (57)	0.63
Enthesitis	5 (10)	15 (8)	0.78

(Adapted from: Rojas-Vargas M et al. First signs and symptoms of spondyloarthritis—data from an inception cohort with a disease course of two years or less (REGISPONSER-Early). Rheumatology (Oxford). 2009;48(4):404–409)

Significance obtained by the chi-square test for contingency tables

Comparison of REGISPONSER-Early (≤2 years) vs REGISPONSER-Late (>10 years)

For proper treatment of PsA, it is necessary to consider all aspects of the disease, including clinical pathology and psychological issues [15]. Further, worse outcomes in patients with PsA are associated with a delay in diagnosis, disability, and joint damage, whereas male sex and lower burden of inflammation at presentation were predictors of improved patient outcomes [17–19]. The presence of specific HLA alleles can also identify patients likely to sustain joint damage [19].

Maintaining good functional status is the primary aim of pharmacologic treatment in patients with PsA. Although the recommended approaches to the diagnosis, therapy, and follow-up of patients with PsA have changed numerous times over the past decade, the goal of treatment is remission or, alternatively, low disease activity or minimal disease activity (MDA) if remission is not attainable [20, 21]. Loss of physical functioning directly impairs patient quality of life (QoL) and results in increased direct and indirect costs associated with the disease [22]. For example, decreased functional status can prevent patients from performing daily activities of living, such as washing and dressing [23].

Clinicians should be aware of the relevant extra-articular manifestations of PsA and its associated comorbidities, which result in considerable morbidity and mortality [24]. At least 1 extra-articular immune-mediated inflammatory disease is present in 94.7% of patients with PsA and the most common of these are psoriasis (94%), uveitis (1.3%), and inflammatory bowel disease (0.7%) [25]. The extra-articular manifestations and comorbidities associated with PsA can also significantly impact QoL and must be considered in the management of PsA [23].

### Risks associated with comorbidities

Patients with PsA have a higher prevalence and incidence of cardiovascular (CV) disease than the general population [24, 26]. This increased risk is due to both a higher prevalence of traditional risk factors such as hypertension, obesity, diabetes, and hyperlipidemia, and also to nonconventional risk factors, for example, those related to chronic systemic inflammation, including higher levels of C-reactive protein and erythrocyte sedimentation rate [24, 27]. Interestingly, suppression of inflammation in patients with PsA has been linked with improvements in surrogate markers of CV risk, such as increased carotid intima media thickness and endothelial dysfunction [28]. Further, the presence of metabolic syndrome and insulin resistance, which are also more common in patients with PsA, are associated with increased severity of PsA and increased risk of CV disease [29, 30]. These findings suggest that systemic inflammation may play an important role in driving CV risk in patients with PsA.

Obesity is reported in 35% of patients with PsA and has been suggested as a potential risk factor for developing PsA [31, 32]. Obesity may also impact disease activity and response to therapy, possibly through increased production of inflammatory cytokines [33]. For instance, irrespective of the kind of therapy used, obesity is associated a lower probability of achieving MDA and it is speculated that the chronic pro-inflammatory state, the biomechanical effect of heavy weight on the joints, and the altered pain threshold associated with obesity may be factors responsible preventing achievement of MDA [32].

PsA is associated with diabetes mellitus and the rate of diabetes mellitus is significantly higher in patients with PsA than in those with RA (odds ratio 1.56; 95% confidence interval [CI] 1.07–2.28; *P* = 0.02) [34]. Caution should be used if prescribing glucocorticoids because they are associated with an approximately 30% increase in the risk for developing diabetes mellitus in patients with psoriasis or PsA [35]. Some dermatologists consider psoriasis as a contraindication for use of corticosteroids, due to concerns of converting plaque psoriasis into pustular psoriasis. However, corticosteroids are still often used across various practice settings, including dermatology.

A strong relationship has been noted between gastrointestinal (GI) inflammation and joint inflammation in various forms of SpA [36]. However, the prevalence of inflammatory bowel disease in patients with PsA is less clearly defined [24]. Of note, there is a significantly increased risk of Crohn’s disease in women with psoriasis (relative risk [RR] 3.86, 95% CI 2.23–6.67) that is further increased in women with both psoriasis and PsA (RR 6.43, 95% CI 2.04–20.32) [37].

Patients with PsA also suffer from sleep disturbances and diminished sleep quality that are associated with generalized pain, anxiety, enthesitis, increased levels of C-reactive protein, and increased erythrocyte sedimentation rate [38]. The prevalence of depression in patients with PsA is 22.2%, compared with 9.6% in patients with psoriasis alone, and the estimated prevalence in the general population (9%) [39]. Similarly, the prevalence of anxiety among patients with PsA is reported as 36.6%, compared with 24.4% in patients with psoriasis alone [39].

### Mitigation of disease-specific risks

Mitigation and evaluation of disease-specific risks is dependent upon prompt and accurate diagnosis. Currently, the primary objectives in clinical rheumatology are early diagnosis and initiation of treatment because diagnostic delays are a significant contributor to poor patient outcomes [4, 15]. Even short delays (6 months) from symptom onset to first visit with a rheumatologist have been observed to contribute to development of peripheral joint erosions and worse long-term physical function [40]. To this end, remission in PsA has been attributed to early diagnosis and treatment.

Disease-related risks should be evaluated through good patient history and use of metrics specific to PsA [41]. These include evaluation of physical function and skin involvement, as well as joint examination (tender joint count, swollen joint count, entheseal assessment). Additionally, physicians should be aware of the possibility of PsA when diagnosing and treating patients who suffer from pre-existing psoriasis. Tracking of metrics over time should be undertaken to detect whether medications are ameliorating disease severity. Imaging may be used for diagnosis and following disease progression over time [41]. In recent years, magnetic resonance imaging (MRI) and ultrasonography have been increasingly used for assessment of PsA and have provided additional information on the pathogenesis of PsA [15].

**Fig. 1:**
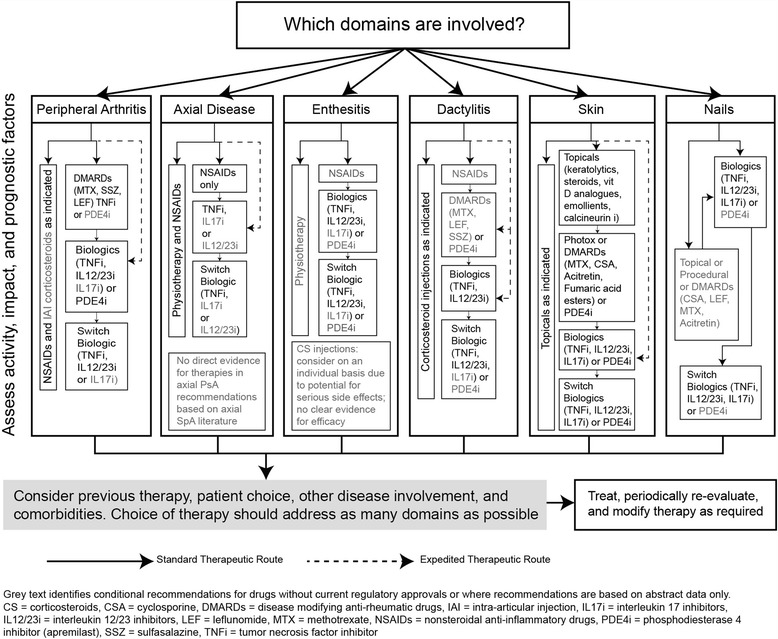
GRAPPA treatment schema for active PsA [16]. Reprinted with permission from: Coates LC et al. Group for Research and Assessment of Psoriasis and Psoriatic Arthritis 2015 treatment recommendations for psoriatic arthritis. Arthritis Rheumatol 2016;68(5):1060–1071

Treatment with drugs is necessary for patients with PsA and recommended treatment guidelines tailored to individual PsA characteristics are shown in Fig. 1 [16]. Although it is important for patients with PsA to remain active, it is typically not possible to manage disease symptoms through physical therapy alone.

## Treatment-related risks

### Risks associated with treatment strategy

Early recognition and intervention with therapy is key to controlling disease progression in patients with PsA [15]. The tumor necrosis factor (TNF) inhibitors etanercept, infliximab, adalimumab, golimumab, and certolizumab pegol have been shown to improve signs and symptoms of PsA [42–46]. Additionally, apremilast (an oral phosphodiesterase-4 inhibitor), abatacept (a T cell selective costimulation modulator), ustekinumab (an interleukin- [IL-] 12/23 inhibitor), and secukinumab (an IL-17A inhibitor) have also shown varying degrees of efficacy in the treatment of PsA [47–53]. Approved agents are discussed in more detail in the following section.

Treat-to-target is a therapeutic concept derived from RA and other diseases [54, 55], which has been proposed for all forms of SpA [20, 41, 54, 56]. With this approach, a clear target, such as remission or low disease activity, is identified and the goal is to sustain this response over time, with an understanding of the need to treat flares and keep tight control of disease activity [55]. A universal definition of the target (e.g., remission, prevention of flare of disease) is required for the treat-to-target approach and, in SpA, remission and sustained low disease activity have been suggested as possible targets [20, 55]. Although definitions of remission or MDA in SpA have been proposed, none have been widely accepted or endorsed and given the multifaceted nature of SpA, a composite of outcome measures may be most useful [54, 57].

The Tight Control of Psoriatic Arthritis (TICOPA) trial, an open-label, randomized, controlled study, has recently provided evidence on the benefit of treating-to-target in PsA [58]. This study compared treat-to-target versus standard of care in newly diagnosed patients with PsA receiving methotrexate (MTX), a combination of disease-modifying antirheumatic drugs (DMARDs), or TNF inhibitors. Results from TICOPA demonstrated that tight control of disease activity using a step-up dosing regimen to achieve MDA (reviewed and adjusted, if necessary, every 4 weeks) significantly improved joint outcomes compared with standard of care (per the treating physician, reviewed every 12 weeks). After 48 weeks, the odds of achieving an American College of Rheumatology (ACR) 20% criteria for improvement (ACR20 response) nearly doubled (odds ratio 1.91; 95% CI, 1.03–3.55; *P* = 0.0392) without any unexpected serious adverse events (AEs) [58].

### Risks associated with pharmacologic treatment

The complexities of PsA pathology suggest the need to identify suitable therapies to address the different combinations of clinical manifestations [21]. Traditional treatments for PsA include nonsteroidal anti-inflammatory drugs (NSAIDs), steroids, and synthetic DMARDs [59]. NSAIDs are effective for relieving musculoskeletal symptoms due to joint inflammation, but have no efficacy on psoriatic skin lesions and are associated with AEs including renal toxicity, gastrointestinal toxicity, and the risk of developing CV events [59]. Traditional DMARD treatment options for PsA include sulfasalazine and MTX [16, 59, 60]. There is debate over the efficacy of MTX and randomized trials have generally been unable to show efficacy [59, 61, 62]. Additionally, MTX is ineffective in treating axial inflammation and there is little evidence to support its use for other symptoms such as enthesitis [59, 62]. However, in the placebo-controlled Methotrexate in Psoriatic Arthritis (MIPA) trial (that showed no benefit with respect to the primary endpoint of PsA response criteria), treatment with MTX resulted in a significant improvement over placebo in both patient and physician global assessments, as well as Psoriasis Area and Severity Index (PASI) scores at 6 months [61, 62]. Nonetheless, the MIPA authors asserted that their findings questioned the classification of MTX as a DMARD in the setting of PsA, with recognition that there are safety concerns associated with MTX therapy [61]. In particular, the use of MTX in obese patients may lead to potential liver damage [63]. Alcohol abuse and the high prevalence of fatty liver in obese patients may contribute to the development of liver damage and the presence of fatty liver can also impede monitoring for liver damage.

Current targeted treatment options approved for PsA include biologics (TNF inhibitors, ustekinumab, and secukinumab) and the small molecule apremilast [3, 59]. These therapies are typically recommended for use after inadequate response to at least one DMARD but early escalation can be considered, especially for patients with poor prognostic factors such as raised inflammatory markers or high active-disease joint counts [16]. For patients who fail a biologic therapy, the GRAPPA guidelines provide a conditional recommendation for switching to a different biologic agent within a drug class or to a drug with a different mode of action [16]. While switching TNF inhibitors can be effective, response rates tend to diminish with successive TNF inhibitor use [64]. There has been investigation of combining TNF inhibitors with a DMARD to prolong biologic persistence but a clear benefit of combination therapy was not observed [65]. There are currently two biologics (secukinumab and ustekinumab) available for treatment of PsA with a different mechanisms of action than TNF inhibition [50, 51, 53]. A concern with all biologic therapies for PsA is an increased risk for infections and patients should be monitored for the development of serious infections that would necessitate discontinuation of treatment until the infection resolves. Reactivation of tuberculosis has been observed with TNF inhibitors and patients must be monitored for active tuberculosis while receiving these agents.

Additionally, response to biologic agents can be limited by immunogenicity and the development of antidrug antibodies [66]. Antidrug antibodies have been observed in 29% of patients receiving adalimumab and 21% of patients receiving infliximab and their presence was significantly correlated with low drug levels and high levels of disease activity [66]. This study also reported that MTX significantly decreased the prevalence of antidrug antibodies, and use of MTX should be considered for patients treated with TNF inhibitors. With secukinumab and ustekinumab, treatment-emergent antidrug antibodies were reported in 0.2% to 0.3% and 9.3%, respectively, of patients from phase 3 trials [49, 52, 53]. In patients receiving ustekinumab and MTX, antidrug antibodies were less common (6.4%) than in those only receiving ustekinumab (12.3%) [52].

Treatment of skin disease associated with PsA can include phototherapy such as ultraviolet B or oral psoralen followed by ultraviolet A (PUVA) [67]. PUVA therapy carries an increased risk of skin cancer compared with other forms of ultraviolet light treatment and patients receiving this treatment should be monitored for skin cancer [67, 68].

### Risks associated with poor compliance

Patient compliance to therapy is an underlying concern in the management of PsA. In patients treated with TNF inhibitors, age has been associated with increased compliance and female sex, comorbidity, and poor clinical condition at baseline have been associated with decreased compliance [69]. Poor adherence can reduce therapeutic efficacy and increase medical costs due to the need for more aggressive treatments [69, 70]. In general, there is the potential for reduced compliance if a patient cannot observe their disease directly, although this is less likely in patients with PsA because skin involvement is more common.

## Psychosocial risks

The impact of PsA is not limited to the skin and joints and patients often experience substantial impairment in QoL [71]. There is an increased risk of depression and anxiety in patients with PsA, which can complicate treatment [71]. Symptoms of depression and anxiety are associated with low treatment adherence in chronic diseases and can impair the ability of patients to self-manage [72]. Physicians should remain cognizant of depressive behaviors such as alcoholism, nonsocial behavior, drug addiction, and suicide ideation and even though treatment of PsA can improve symptoms of depression and anxiety, it is still important for physicians to identify individuals who would benefit from referral for counseling.

Patients with both PsA and psoriasis experience worse QoL than those with only psoriasis [73]. Additionally, this difference in QoL was not due to differences in other comorbidities between patients with and without joint involvement [73]. Collectively, further efforts should be made by physicians to identify patients with PsA who should be referred for counseling as well as to monitor changes in these psychosocial issues over the course of treatment.

## Conclusions

Among patients with PsA, a major emphasis of comprehensive care should be aimed at mitigating risks and improving physical health-related QoL. As outlined above, this may be achieved by early diagnosis, prevention of disease progression, treatment of PsA-associated physical morbidity, mitigation of treatment-related risk, and treatment of associated medical comorbidity. Various agents, including newer biologics, have approved indications for use in the PsA population—providing the clinician and patients with choices of agents based on their specific disease symptoms. Overall, management of patients with PsA is complex and requires the adoption of a more patient-focused multidisciplinary approach.

## Abbreviations

ACR: American College of Rheumatology
AEs: Adverse events
CASPAR: ClASsification criteria for Psoriatic Arthritis
CI: Confidence interval
CV: Cardiovascular
DIP: Distal interphalangeal
DMARDs: Disease-modifying antirheumatic drugs
GI: Gastrointestinal
GRAPPA: Group for Research and Assessment of Psoriasis and Psoriatic Arthritis
HLA: Human leukocyte antigen
IBD: Inflammatory bowel disease
IL: Interleukin
MDA: Minimal disease activity
MIPA: Methotrexate in Psoriatic Arthritis
MRI: Magnetic resonance imaging
MTX: Methotrexate
NSAIDs: Nonsteroidal anti-inflammatory drugs
PASI: Psoriatic Area and Severity Index
PsA: Psoriatic arthritis
PUVA: Psoralen followed by ultraviolet A
QoL: Quality of life
RA: Rheumatoid arthritis
RR: Relative risk
SI: Sacroiliac
SpA: Spondyloarthropathies
TICOPA: Tight Control of Psoriatic Arthritis
TNF: Tumor necrosis factor
